# Unveiling pathways: exploring chronological and maturational biases in a Spanish professional soccer academy

**DOI:** 10.3389/fspor.2025.1476448

**Published:** 2025-02-20

**Authors:** Saúl Peñín-Grandes, Silvia Mesonero-García, Florentino Huertas Olmedo, Honorato Jose Ginés Cabeza, Rafael Ballester Lengua

**Affiliations:** ^1^ Research, Development and Innovation Department, Real Valladolid CF, Valladolid, Spain; ^2^ “i+HeALTH” Strategic Research Group, Department of Health Sciences, Miguel de Cervantes European University (UEMC), Valladolid, Spain; ^3^ Department of Physical Education and Sport Sciences, Catholic University of Valencia “San Vicente Martir”, Valencia, Spain

**Keywords:** relative age effects, maturation, football, youth athlete, talent development

## Abstract

This brief research report aimed to analyze the prevalence of asymmetries in players' date of birth, the influence of relative age affects (RAEs) and maturation on players' participation, and the potential maturity biases in performance indicators in a Spanish professional male soccer academy. Maturity status was determined as the percentage of predicted adult height (%PAH). RAEs are strongly represented in the sample as 77% of players were born in the first semester of the year. Relatively older players do not appear to receive significantly more playing minutes, while more mature players showed a higher percentage of playing time in U13 and U14, with no differences in U15 and U16. In other performance indicators, more mature players do not have an advantage when it comes to being considered “promising players”, training and competing with higher chronological age groups and being selected by state teams. Finally, individual maturity level within the team does not appear to be relevant to the club's proposal to continue in the academy. Our results show a strong overrepresentation of players born in the first semester of the year, indicating the presence of RAEs. However, this bias does not translate into significant differences in competitive performance, such as playing time. Conversely, individual maturity status demonstrates a transient effect on playing time in younger age groups (U13 and U14). These findings underscore the importance of understanding the interplay between RAEs and maturity biases in talent identification and development in the highly competitive framework of a professional soccer academy.

## 1 Introduction

Traditionally, the process of talent identification and development in soccer comprises two key stages: (i) talent identification (TID) (i.e., the selection of players who have the potential to develop into elite soccer players), and (ii) talent development (TD) (i.e., the generation of a suitable learning environment for players to develop their full potential) (1). However, this process can be more nuanced, involving additional steps that may overlap or evolve depending on the context (2, 3). The complex process of recognizing, developing, and progressing youth soccer players through elite academies to professional soccer players has been improved over the last decade and there has been an increasing emphasis on the use of science-based development systems that offer a more holistic approach to TID and TD (4). However, this process is still very much linked to the subjective perception of the professional decision makers (i.e., academy scouts, coaches, coordinators and directors) (5) and in soccer, has been characterized by chronological and maturity biases in TID and TD (6).

In this context of recognizing and developing talent, it has been widely reported in the literature that growth-related biological changes and differences in players' date of birth in relation to a cut-off date—also known as relative age effects (RAEs)—are causal factors in the overrepresentation of players who are either relatively older and/or more mature (MM) in elite soccer academies (7–9). In any case, these players are often characterized by a temporary improvement in anthropometric (e.g., stature and body mass) and physical performance parameters (e.g., power, strength, speed) compared to late-maturing and/or late-born players (10) who are often less physically developed (11). This can result in a (un)conscious decrease in their competitive playing opportunities, jeopardizing continuity in elite soccer academies and, consequently, at the expense of the pursuit of short-term performance, hindering their long-term development prospects (12).

However, current evidence from elite soccer academies examining the combined impact of RAEs and biological maturation on competitive participation, evaluation, and retention decisions remains limited. Most existing studies either focus on the distribution of RAEs and maturation within elite academies (13, 14) or investigate impact on physical performance (15–18). While some research addresses how RAEs alone influences player development trajectories (19), no studies, to our knowledge, explore the combined effects of RAEs and maturation on player competitive participation, evaluation, and retention.

The present study offers a novel contribution by analyzing the interaction between RAEs and biological maturation through unique access to internal data from a professional soccer academy. This data provides insights into player participation, growth, and development in a high-performance environment that are difficult to obtain due to the proprietary nature of such information. By examining these variables together, our study sheds light on how developmental and competitive dynamics interact in shaping talent management decisions in a professional academy.

Indeed, the present study was conducted directly by the Research, Development, and Innovation Department of the academy of a professional club within the Spanish First Division League in the 2022–2023 season (La Liga EA Sports) with the main aim to investigate how present chronological and maturational biases were in TID and TD. For this purpose, it was analyzed: (i) the prevalence of asymmetries in players' date of birth; (ii) the influence of chronological age and maturation on players' competitive participation and; (iii) the potential physical and maturity biases in: (a) the consideration as a “promising players” (i.e., rated by the club with higher projection to become professionals), (b) the call-up for state teams and (c) proposal of continuity/release within the club (i.e., whether the players continue their development in the academy or not).

## 2 Material and methods

### 2.1 Sample

The present study was conducted in the academy of a professional club of the Spanish First Division League in the 2022–2023 season (LaLiga EA Sports). A total of 80 male soccer players were categorized according to their chronological age (U-13, *n* = 21; U-14, *n* = 18; U-15, *n* = 22; U-16, *n* = 19). Parents and players signed agreements for the use of their data for internal and external research purposes of the club. Additionally, parents completed a form including the parents' height. The present study was approved by Catholic University of Valencia Research Ethics Committee (UCV/2019–2020/149) in accordance with the Declaration of Helsinki.

### 2.2 Data collection and procedures

#### 2.2.1 Chronological and relative age

The youth academy leagues within the Spanish Soccer Federation are currently organized according to the players' chronological year (cut-off date of January 1st). One member for the Research, Development and Innovation Department of the club collected information on the birth date of each player and grouped according to (i) the birth quarter: Birth Quarter 1 (Q1), January to March; Q2, April to June; Q3, July to September; and Q4, October to December; (ii) the semester: first semester, January to June; and second semester, July to December.

#### 2.2.2 Anthropometric characteristics

Anthropometric characteristics were collected at three points in time during the 2022–2023 season: measurement 1, in September 2022; measurement 2, in January 2023; and measurement 3, in June 2023. All three measurements were applied to all players within the same week in each of the measurement time points. The players were tested 30 min before starting the training session under standardized conditions (18 ± 2° C) at the club's sports facilities by an expert from the club's Health & Performance Department. Standing and sitting height were measured with a Seca 213 stadiometer (“Physical distancing for health”, Hamburg, Germany) with an accuracy of 0.1 cm according to the ISAK protocol (International Society for the Advancement of Kinanthropometry) (20). Body weight was determined with a Tanita BC-602 scale (Tanita Europe B.V., Amsterdam, Netherlands) with an accuracy of 0.1 kg, with the players wearing their training clothes (socks, t-shirt, and shorts) (21). Height and weight were measured twice. If height and body mass differed by more than 0.4 cm or 0.4 kg respectively, a third measurement was taken, and the mean value was calculated (22).

#### 2.2.3 Maturity status

The Percentage of Predicted Adult Height (%PAH), measured with the Khamis-Roche (KR) method, has been shown to be a reliable tool for assessing the maturation status of soccer players (23). This method is based on the use of a predictive equation that requires precise anthropometric measurements, including the standing height (cm/in) and body mass (Kg/lb) of the soccer player, as well as the standing height (cm/in) of both parents (24). A formula is applied, considering the reference coefficients provided by Khamis and Roche: %PAH = *β*0 + [*β*1 + standing height (cm/in)] + [*β*2 × weight (lb/kg)] + [*β*3 × mean standing height of both parents (cm/in)] (24). In this formula, *β*1, *β*2, and *β*3 are the coefficients established in the reference table provided by Khamis and Roche, while *β*0 is the smoothed regression coefficient for boys and girls within the KR method (24). The obtained results are compared with the reference table to estimate the %PAH in which the subject falls (25). As the height of parents was provided by themselves, a correction adjustment for overestimation has been carried out according to the criteria by Epstein et al. (26).

To determine the maturation stage of each team's players, they were classified into two maturity groups—Less Mature (LM) and MM—based on the %PAH measured at the beginning of the season (Table 1). This classification was conducted using the median %PAH value within each specific age group as the cut-off point. Players with %PAH values below the median were classified as LM, while those above were categorized as MM. This method ensures relative comparisons of biological maturity within each age group, resulting in two groups of equal size. For instance, in the U14 category, the %PAH ranged from 87.98% to 95.02%, with a median cut-off of 90.2%, classifying nine players as LM and nine as MM.

**Table 1:** Anthropometrics characteristics and playing time depending on birth quarter in different age groups.

Age group	Q1				Q2				Q3				Q4				Height		Weight		PT	
	*n*	H (cm)	W (Kg)	PT (%)	*n*	H (cm)	W (Kg)	PT (%)	*n*	H (cm)	W (Kg)	PT (%)	*n*	H (cm)	W (Kg)	PT (%)	*F*	*p*	*F*	*p*	F	*p*
U-13_(*n*_ _=_ _21)_	10	157.5 (±6.2)	44.5 (±6.9)	67.5 (±17.1)	5	150.1 (±8.0)	39.0 (±3.9)	69.1 (±12.1)	0	–	–	–	6	148.4 (±1.0)	40.5 (±3.8)	53.6 (±7.0)	5.571	**.013**	1.930	.174	1.282	.301
U-14_(*n*_ _=_ _18)_	6	160.1 (±7.0)	48.8 (±8.6)	58,0 (±20.0)	8	164.3 (±9.2)	51.0 (±9.1)	65.2 (±19.0)	2	158.9 (±0.6)	46.6 (±5.3)	63.2 (±34.4)	2	160.8 (±14.1)	48.6 (±14.3)	60.7 (±1.1)	0.380	.769	0.156	.924	0.149	.929
U-15_(*n*_ _=_ _22)_	9	169.8 (±8.9)	60.4 (±8.9)	67.5 (±19.0)	10	168.3 (±7.3)	60.0 (±6.3)	64.5 (±18.6)	3	166.7 (±11.0)	57.8 (±8.6)	84.2 (±19.7)	–	–	–	–	0.165	.849	0.127	.881	1.282	.301
U-16_(*n*_ _=_ _19)_	10	174.0 (±5.0)	65.5 (±7.2)	71.9 (±21.6)	3	171.8 (±(5.1)	62.5 (±6.9)	65.0 (±8.7)	4	178.0 (±7.3)	70.8 (±3.3)	52.3 (±13.2)	2	170.2 (±12.1)	58.5 (±8.7)	97.2 (±1.8)	0.923	.454	1.764	.197	2.898	.070

Q, Birth Quarter; *n,* number of player per group; H, Height; W, Weight; PT, Playing time; *F*: Anova Value, *p*: significance *p*-value.

Mean and SD (between parenthesis) values.

Bold values denote statistical significance at the *p* < .05 level.

Research has consistently shown that elite academy players tend to exhibit greater biological maturity compared to their non-elite counterparts (7). In line with previous research (27), grouping players by their relative maturity within each age group—rather than applying absolute classifications such as “early” or “late” maturers based on the general population—provides a contextually relevant framework that reflects the competitive dynamics of professional academy teams and supports a more nuanced analysis of the relationship between maturity and performance.

#### 2.2.4 Players' performance indicators

Players' performance indicators were defined according to the number of participations in competition, their consideration as “promising players”, the call-up for state teams and proposal of continuity/release within the club. Participation in regular competition in 2022–2023 season was assessed by the coaching staff weekly, based on the total minutes played in official competitions by each player according to their chronological age team. Participation was expressed as a percentage (%) of the total minutes in which the player was available (i.e., had no injuries or could not compete for non-sporting causes). For example, a player who played 45 out of 90 min (total time) in five games would be given a percentage of 50% of playing time. Moreover, within the academy, players identified as having high potential to become professionals were classified as “promising players”. This classification was based on a structured qualitative assessment carried out by the academy's performance department in line with the club's philosophy. Players were assessed on four key dimensions: (i) technical, (ii) tactical, (iii) physical and (iv) psychological using an A, B, C or D standardized scale. Based on these assessments, players were categorized into four levels: A (“Promising player”—high chance to become professional), B (moderate chance), C (low chance) and D (no chance). There was no set limit on the number of players in each category. Many of these promising players were training and competing with older age groups.

Every call-up of a player to the state soccer team for official matches or tournaments were registered by the person responsible at the academy and integrated into a database maintained by the club specifically for this purpose. Additionally, at the end of the 2022–2023 season, the decision to retain or release each player was based on a proposal made by different academy professionals, following a thorough qualitative assessment. This recommendation was officially recorded by the academy director, independent of the player's own decision to stay or leave the club. This measure is significant as it reflects the club's long-term investment strategy and ensures that players who remain in the system have been identified as promising based on multiple professional perspectives.

#### 2.2.5 Statistical analysis

The examination of birth quarter distribution (Q1, Q2, Q3, and Q4) in conjunction with player maturation levels (LM and MM) was carried out using a frequency table. This involved adjusting through chi-square goodness-of-fit tests to assess homogeneity and an analysis of differences between expected and observed frequencies. For the birth quarter, we adopted the assumption of 25% for each birth quarter, consistent with previous studies (28) and the Spanish population census data for individuals born between 2007 and 2010 (Instituto Nacional de Estadística, INE). As the chi-square test alone does not reveal the magnitude and direction of the relationship, therefore, a odds ratio (OR) analysis and 95% confidence intervals (CIs) were included, comparing the results between Q1 and Q4. The OR indicates the probability of membership; a result of 1.0 suggests equal group membership probability, while 2.0 indicates double the probability of one group compared to the other (29). Regarding maturation, cut off values of 50% within the chronological age group were used to determine whether players were relatively LM or MM compared to their peers (30).

Considering that the Kolmogorov–Smirnov and Levene tests indicated normal distribution and homogeneity of variance (*ps* < .05) for the *Playing minutes* variable, group comparisons based on relative age (Q) were conducted using ANOVAs. Comparison between the two maturation groups were analyzed through independent samples *t*-test. Effect sizes were reported using Cohen's *d*, classified as small (*d* = 0.2), medium (*d* = 0.5), and large (*d* ≥ 0.8) (31).

Correlation between the athlete's %PAH and minutes played was assessed using Pearson correlation. Correlation coefficients below 0.2 were considered very low, between 0.21 and 0.40 were categorized as low, between 0.41 and 0.60 as moderate, from 0.61 to 0.8 as high, and above 0.8 as very high (32). All statistical analyses were performed using IBM SPSS Statistics software (Version 25.0, Chicago, IL, USA).

## 3 Results

### 3.1 Relative age effect and maturation

**Figure 1:**
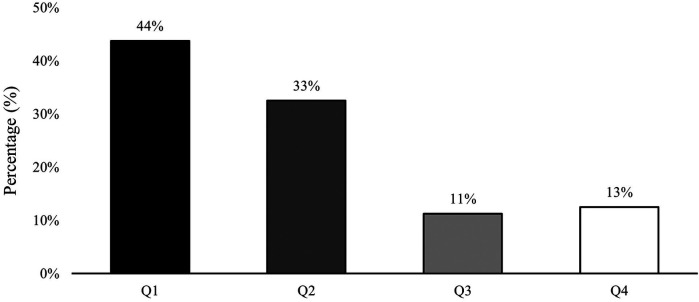
Distribution of players by birth quarter in the academy. Q, Birth Quarter.

**Figure 2:**
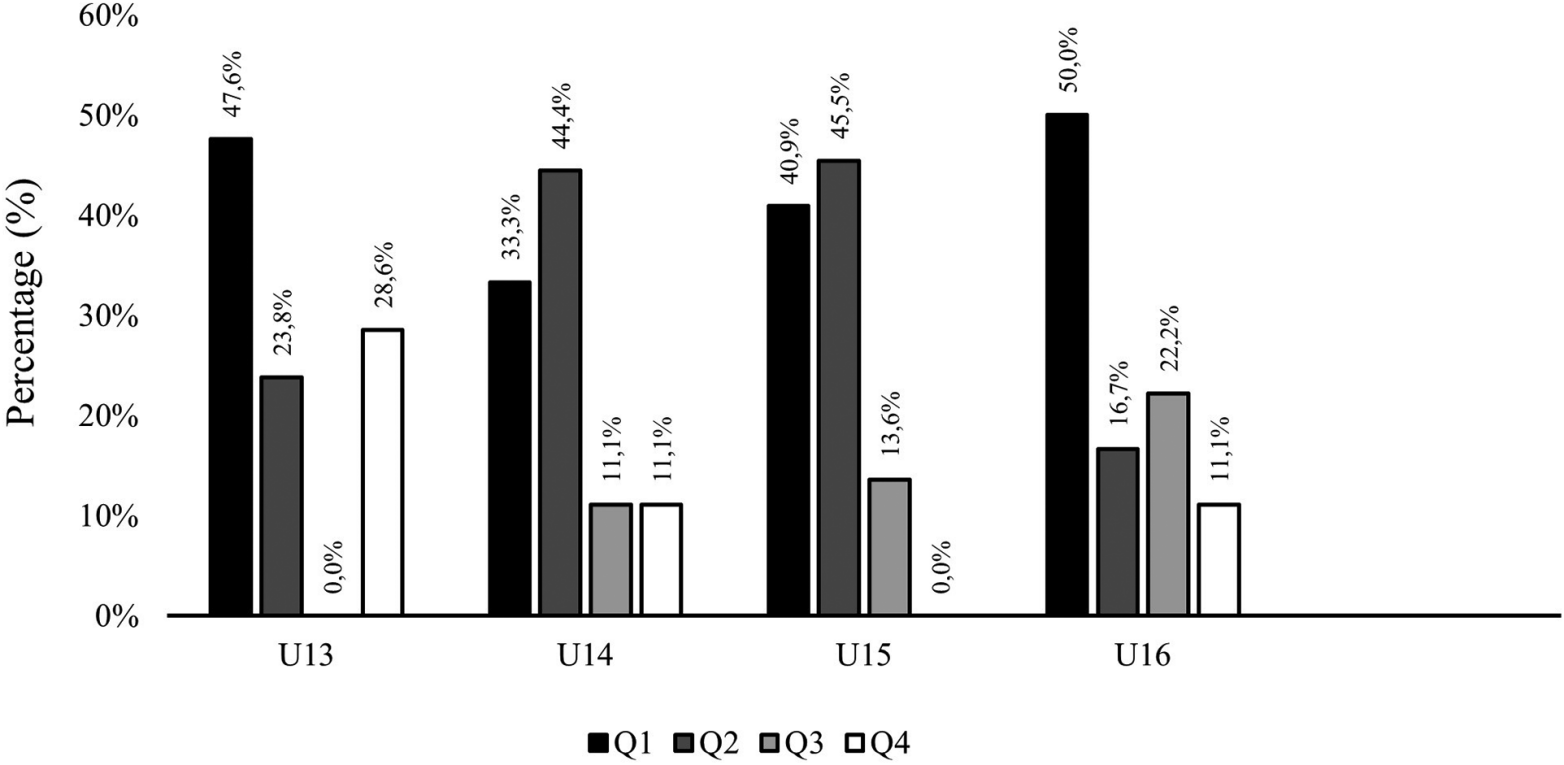
Distribution of players by birth quarter across the age groups. Q, Birth Quarter.

The results of Birth Quarter (Q) distribution in the total sample are depicted in Table 1 and Figure 1 (total sample), and Figure 2 (RAEs per age group). RAEs were observed in this soccer academy [*χ*^2^ (3, *N* = 80) = 24.100; *p* < .001], showing a 77% of players born in the first semester, and obtaining an OR = 3.5, IC (1.34–9.14) when comparing players born in Q1 with those born in Q4. In terms of age group results, the U13 and U15 groups did not include players born in Q3 or Q4, respectively. In the U14 group, no significant differences were noted according to birth quarter [U14: *χ*^2^ (3, *N* = 18) = 6.000; *p* = .112; OR = 3.0, IC (0.40–22.71)]. However, the U16 group showed significant differences favoring players born in Q1 [U16: *χ*^2^ (3, *N* = 19) = 8.158; *p* = .043; OR = 5.0, IC (0.64–39.06)].

**Figure 3:**
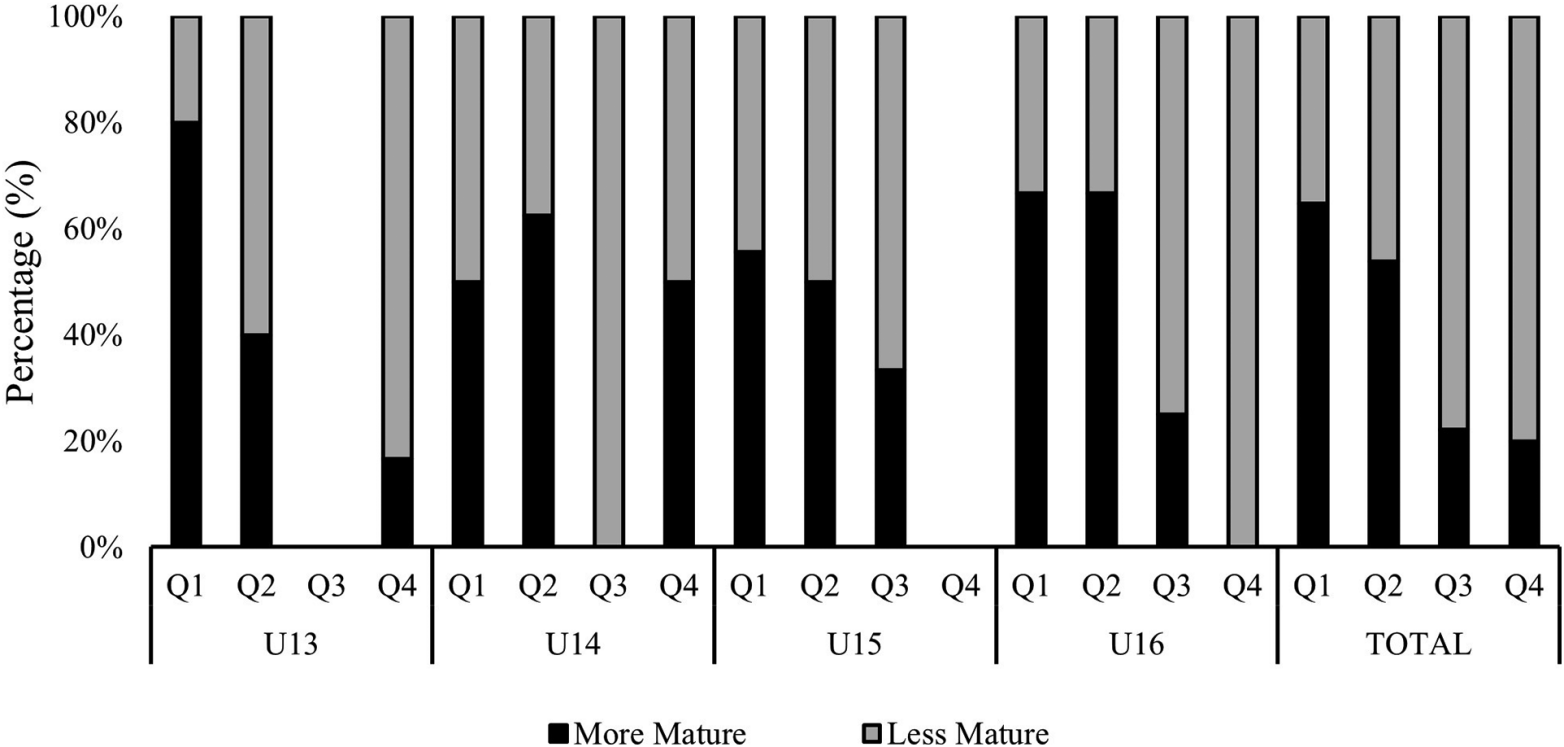
Distribution of maturation status by birth quarter across the age groups. Q, Birth Quarter.

When presenting the results regarding the distribution of maturation across birth quarters, the data showed that players born in the first two quarters of the year were predominantly MM compared to those born in the last two quarters of the year (Figure 3). Conversely, there was a higher proportion of LM players among those born in the third and fourth quarters. These results suggest an uneven distribution of maturation across the year, with players born earlier being more biologically advanced. Statistical analysis supports this conclusion [*χ*^2^ (3, *N* = 80) = 9.945, *p* = .019], indicating a significant association between birth quarter and maturity status.

### 3.2 Relative age effects, maturation and playing time

Regarding the influence of relative age on players' individual performance indicators, our results indicate that playing time was not influenced by the birth quarter [*F*(3, 76) = 0.098, *p* = .961] in none of the age groups (Table 1).

**Figure 4:**
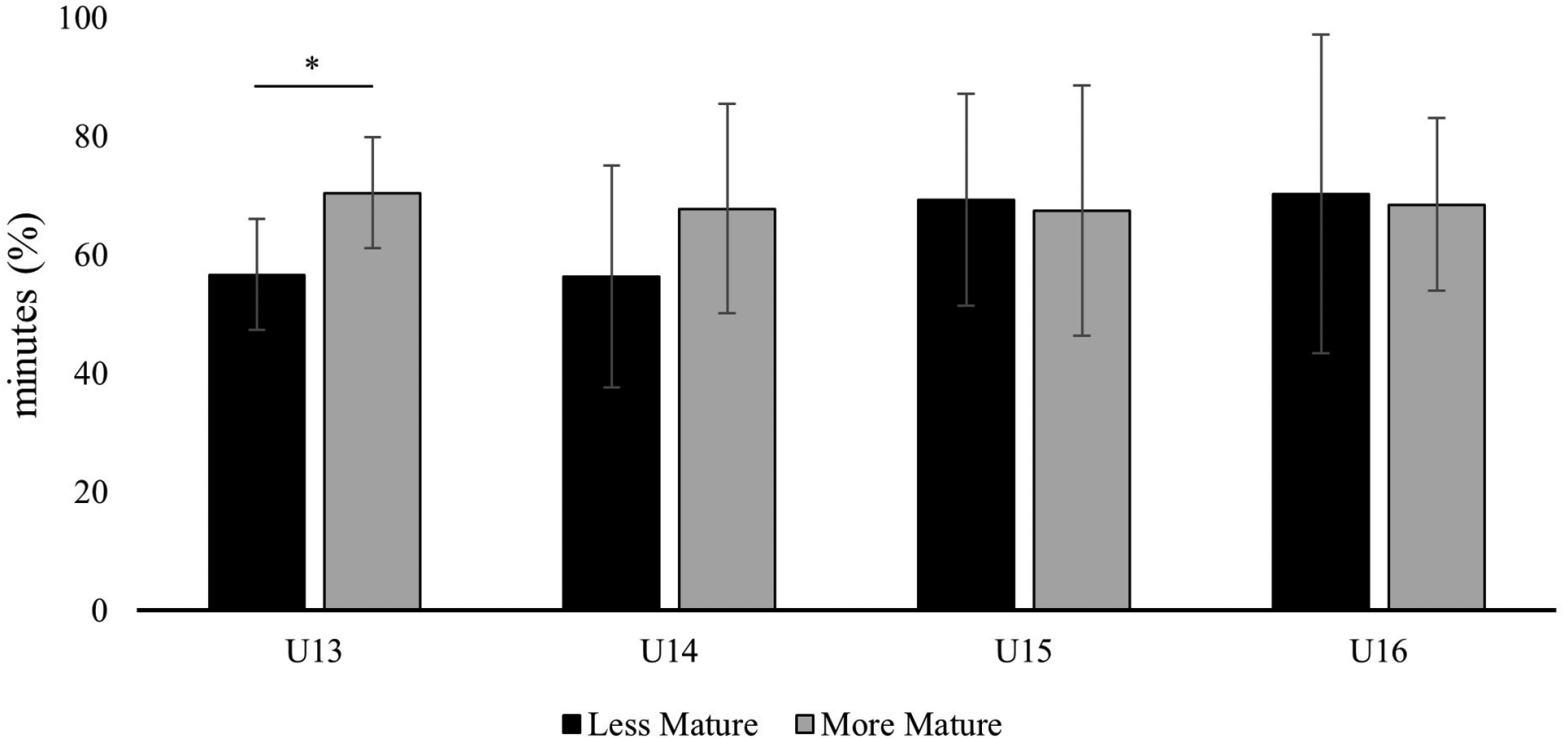
Distribution of playing time by maturation status across age groups. *p *<* *.05*.

Analyzing the distribution of minutes based on players' maturation, Table 2 and Figure 4 reveals that MM players (*M* = 68.6, *SD* = 16.9) played more percentage of minutes than their LM counterparts (*M* = 63.3, *SD* = 19.4), although these differences did not reach statistical significance [*t*(78) = −1.29; *p* = .199], due to the high variability within each group and sample size. However, upon closer examination of distinct age categories, it was found that the youngest players (U13 and U14), classified as LM, experienced over 12% less playing time compared to their MM counterparts. The disparities in the U13 group were statistically significant [*t*(19) = −2.36; *p* = .029]. Indeed, moderate significant correlations between maturation and playing minutes were observed both in the U13 and U14 categories [U13: *r*(21) = .545, *p* = .011; U14: *r*(18) = .573, *p* = .013], whereas the older groups did not exhibit significant correlations [U15: *r*(22) = −.142, *p* = .527; U16: *r*(19) = −.107, *p* = .664]. The interaction between birth quarter and maturity level did not yield significant results, indicating that the effect of birth quarter on playing time does not vary according to players' maturity status [*F*(3, 72) = 0.248, *p* = .862].

**Table 2:** Anthropometrics characteristics and playing time depending on the maturation in different age groups.

Age group		Less mature					More mature					Height		Weight		%PAH		PT	
	%PAH	*n*	H (cm)	W (Kg)	%PAH	PT (%)	*n*	H (cm)	W (Kg)	%PAH	PT (%)	*t*	*p*	*t*	*p*	*t*	*p*	*t*	*p*
U-13_(*n*_ _=_ _21)_	82.54–89.87	10	148.2 (±4.5)	38.9 (±3.7)	83.8 (±0.9)	56.7 (±9.4)	11	157.6 (±5.8)	44.9 (±6.1)	87.5 (±1.3)	70.5 (±16.1)	−4.08	**<.001**	−2.68	**.015**	−7.56	**<.001**	−2.36	**.029**
U-14_(*n*_ _=_ _18)_	87.98–95.02	9	155.7 (±3.8)	42.8 (±4.9)	89.1 (±0.8)	56.4 (±18.7)	9	168.1 (±6.3)	56.1 (±5.4)	92.9 (±1.7)	67.8 (±17.7)	−5.06	**<.001**	−5.49	**<.001**	−5.79	**<.001**	−1.32	.206
U-15_(*n*_ _=_ _22)_	88.46–97.40	11	164.9 (±9.0)	56.0 (±6.4)	93.6 (±2.0)	69.3 (±17.9)	11	172.5 (±5.2)	63.7 (±6.5)	96.5 (±0.5)	67.5 (±21.1)	−2.40	**.029**	−2.84	**.010**	−4.70	**<.001**	0.21	.836
U-16_(*n*_ _=_ _19)_	95.82–99.10	10	172.6 (±8.3)	63.9 (±8.8)	96.7 (±0.8)	70.3 (±26.9)	9	175.4 (±3.3)	66.8 (±5.3)	98.2 (±0.5)	65.5 (±14.6)	−0.96	.351	−0.87	.394	−4.89	**<.001**	0.19	.855

%PAH, Percentage of Predicted Adult Height; *n*, number of player per group; H, Height; W, Weight; PT, Playing time; *t*, *t*-Student; *p*, significance *p*-value; “Less Mature” (<50%), “More Mature” group (>50%).

Bold values denote statistical significance at the *p* < .05 level.

### 3.3 Relative age effects, maturation and anthropometric parameters

RAEs analysis revealed no significant differences (*ps* > .05) in anthropometric variables when comparing birth quarters across the different age groups (Table 1). However, MM players showed higher anthropometric parameters (height and weight) than their LM teammates, with significant differences (*ps* < .05) in the U13, U14 and U15 groups (Table 2).

### 3.4 Relative age effects, maturation and players' projection

As shown in Table 3, the analysis of RAEs revealed significant differences in player continuity (*p* < .001) whereas these differences did not reach statistical significance for promising and promoted players (both *ps* = .062). This pattern is consistent with the greater representation of players born in the first two quarters of the year in the sample. Given this distributional bias, it is expected that similar trends will emerge for these variables as the overrepresentation of players born early influences the observed results.

**Table 3:** Players' projection based on maturation and RAEs.

	Less mature		More mature		*χ* ^2^	*p*	Q1		Q2		Q3		Q4		χ^2^	*p*
	*n*	%	*n*	%			*n*	%	*n*	%	*n*	%	*n*	%		
Player continuity	34	47.9	37	52.1	0.127	.722	32	45.1	22	31.0	7	9.9	10	14.0	22.352	**<** **.** **001**
Promising players	8	44.4	10	55.6	0.222	.637	9	50.0	5	27.8	2	11.1	2	11.1	7.333	.062
Promoted players	8	42.1	11	57.9	0.474	.491	9	47.4	6	31.6	2	10.5	2	10.5	7.316	.062

*χ*^2^, Chi-square; *n*, number of player per group; *p*, significance *p*-value.

Bold values denote statistical significance at the *p* < .05 level.

Regarding the maturation analysis, 47.8% of the players who were offered the opportunity to continue within the club belonged to the LM group, while 52.2% belonged to the MM group, though this difference was not statistically significant, [*χ*^2^(1, *N* = 71) = 0.127; *p* = .722]. Among the players considered as more promising within the club, 44.4% belonged to the LM group while 55.6% were included in the MM group. Similarly, from the group of players promoted to older teams within the club, 42.1% belonged to the LM group and 57.9% to the MM group. In none of these previous comparison significant differences were found [*χ*^2^ (1, *N* = 18) = 0.222; *p* = .637 and *χ*^2^ (1, *N* = 19) = 0.474; *p* = .491, respectively]. Finally, regarding the players selected for the state team, 40.0% of them were LM players, while the remaining 60.0% belonged to the MM group, although again these differences did not reach statistical significance [*χ*^2^ (1, *N* = 10) = 0.400; *p* = .527].

## 4 Discussion

This study aimed to analyze: (i) the prevalence of asymmetries in players' date of birth; (ii) the influence of chronological age and maturation in players' competitive participation and (iii) the potential physical and maturity biases in: (a) the proposal of continuity/release within a professional club, (b) the consideration as a “promising players” and (c) the call-up for state soccer teams.

RAEs are strongly represented in the sample examined in this study, which is consistent with previous studies on elite youth soccer academies (9, 16, 19, 33–37). Specifically, 77% of the sample analyzed were born in the first semester of the year. Furthermore, as expected, players born in the first semester were mostly MM, while those born in the second semester had considerable higher proportion of LM players (78% in Q3, 80% in Q4). In this context, when considering the influence of RAEs on players' individual performance indicators, it was found that players born in the first semester of the year received slightly more playing time, with no significant differences between quarters, which is consistent with previous research in Spanish elite soccer academies (38).

In terms of player maturity, the MM players received slightly more playing time than their LM counterparts, although there were no significant differences, as when looking at the whole sample, which is in line with more recent approaches (27, 39).

Similarly, the interaction between maturity and RAEs were not significant, suggesting that maturity status does not differentially impact playing time based on players' born in the different birthquarters. This outcome may reflect the academy's prioritization of competitive balance over physical maturity or relative age. In fact, the lack of significant differences in the percentage of playing time between early and late maturing players is in line with the recommendations of previous authors suggesting that playing decisions should not be based on physical and momentary performance parameters, but also on other performance indications such as technical skills, motor coordination or tactical understanding (40). However, upon further examination within each age category, a moderate correlation between maturity status and playing time was observed in U13 and U14, with MM players showing a higher percentage of minutes played, while there was no correlation in U15 and U16. This result may be evident in the U13 and U14 groups as the differences in maturity levels are higher in this age period (41). Players with early maturity levels have shown in previous studies better aerobic capacity, speed, strength and power than their late maturity counterparts (42, 43). In fact, studies in youth soccer have shown that late-maturing players are consistently excluded as the age- and sport-specific demands increase, especially in this critical phase of adolescence (44).

Nevertheless, our results suggest that MM players do not have a significant statistical advantage in performance/projection indicators such as promotion to higher chronological age categories, being classified as “promising players” within the academy, or selection for state soccer teams. However, it is important to interpret these findings with caution, as the number of players classified as “promising” (*n* = 18), promoted to higher age groups (*n* = 19), or selected for the state team (*n* = 10) was relatively small. Furthermore, in all cases, MM players within their age group were more represented. The lack of significant differences might be explained by this small sample, as well as other contextual factors within the academy's selection processes which prioritize game understanding and technical-tactical skills over anthropometrics and physical performance. This is important to consider as growth and maturation have been shown to influence coaches' and decision makers' perceptions of ability/potential and performance (45), with early maturing players in elite academies being perceived as more capable (6) and with greater potential (46), which may also lead to them being promoted in the academy and called up by their state or national team (47). Finally, in the same vein, it seems that the level of maturity has not been very influential on the club's proposal to remain in the academy. This is an important factor, as physical advantages related to age and/or maturation during adolescence are highly transitory and tend to disappear or even reverse in adulthood, where technical, tactical and cognitive skills -that are prevalent among talented players in the academy- play a key role (10).

We believe that these results are promising for advancing TID and and TD practices, especially as they suggest that it is possible to mitigate the typical effects of RAEs and maturational biases on performance outcomes in a high competitive environment. This may be because the academy has deliberately designed its development criteria to prioritize long-term potential over immediate physical advantage, reducing the biases associated with age and maturity. In addition, awareness-raising activities have been implemented to actively minimize this bias and ensure a fairer and more informed development process. This approach, in line with the club's philosophy, emphasizes the holistic development of players by prioritizing technical and tactical skills and understanding of the game over short-term physical attributes.

There are some limitations that need to be considered in the present brief research report. Our study offers concise and focused findings, reflecting the specific context of a Research, Development, and Innovation Department of the academy of a professional club. Only one team was analyzed for each level of competition, so possible conclusions should be taken with caution as variables could be influenced by contextual factors. While it presents initial data and specific advancements, it is inherently limited in terms of the generalizability of the results due to the limited sample size. Another important limitation is that the maturity was assessed indirectly, To increase accuracy, future research should incorporate direct and validated methods of assessing biological maturation whenever possible, such as radiographic analysis (e.g., x-rays), growth velocity measurements, ultrasound imaging, dual-energy x-ray absorptiometry (DXA), or magnetic resonance imaging (MRI) (48). The use of these direct techniques could provide a more accurate understanding of the maturation process and its potential influence on the variables under investigation compared to indirect approaches (49). Finally, it must be considered that “promising players”, who were estimated with higher prognosis of becoming a professional, have been evaluated by the club's professionals with a subjective nature according to the club's philosophy. For these reasons, future studies should consider increasing the sample size and studying how age, maturation, and physical biases may cross-sectionally and longitudinally influence participation and selection process in other professional soccer academies. The methodological strengths of this study should also be highlighted. Specifically, access to a professional soccer academy. This access allowed for a comprehensive assessment of potential chronological and maturational biases in various performance indicators and provided valuable insight into TID and TD in soccer.

## 5 Summary

RAEs are strongly represented in the sample, as 77% of players were born in the first semester of the year. Relatively older players do not appear to receive significantly more playing minutes, while MM players show a higher percentage of playing time at U13 and U14, with no differences at U15 and U16. In other performance indicators, MM players have no advantage when it comes to being considered “promising players”, training and competing with higher chronological age groups, and being selected by state teams. Finally, individual birth date and maturity level within the team do not appear to be relevant to the club's proposal to continue in the academy. Individual differences in date of birth and biological maturation pose numerous challenges for both players and professional decision-makers at elite soccer academies. It is therefore crucial to understand how these differences can influence TID and player development within the very demanding competitive framework of the professional soccer academies.

## Data Availability

The raw data supporting the conclusions of this article will be made available by the authors, without undue reservation.
